# Ultrasonography of Parasitic Diseases in Domestic Animals: A Systematic Review

**DOI:** 10.3390/ani12101252

**Published:** 2022-05-12

**Authors:** Andrea Corda, Francesca Corda, Valentina Secchi, Plamena Pentcheva, Claudia Tamponi, Laura Tilocca, Antonio Varcasia, Antonio Scala

**Affiliations:** Department of Veterinary Medicine, University of Sassari, 07100 Sassari, Italy; andreacorda@uniss.it (A.C.); plamenapentcheva@gmail.com (P.P.); cltamponi@uniss.it (C.T.); tiloccalaura@yahoo.it (L.T.); varcasia@uniss.it (A.V.); scala@uniss.it (A.S.)

**Keywords:** ultrasonography veterinary parasitology, ultrasound parasitic diseases, ultrasound, parasites domestic animals, helminths, diagnostic imaging

## Abstract

**Simple Summary:**

The aim of this literature review was to summarize the current knowledge on the application of ultrasonography in diagnosis, staging and monitoring of helminthic diseases in domestic animals. We selected and analyzed 122 papers about the application of ultrasonography in parasitic disease in ruminants, equids, swine, and companion animals. Helminths can be recognized in ultrasound images by their morphology, size, and location. In some cases, the parasites are not directly seen by ultrasound, but the lesions caused by them can be easily visualized. Ultrasound imaging is taking on an increasingly important role in veterinary parasitology.

**Abstract:**

The aim of this review was to summarize the current knowledge on the application of ultrasonography in diagnosis, staging and monitoring of helminthic diseases in domestic animals. Only peer reviewed papers written in English language were included in this systematic review. All papers concerning unicellular parasites, wild animals, non-domestic experimental animals, or ex vivo or in vitro applications of ultrasonography were excluded from the review. A total of 122 papers met the inclusion criteria. Among them 47% concerned nematodes, 37% cestodes, and 16% trematodes with the genus *Dirofilaria*, *Echinococcus*, and *Fasciola* the most represented, respectively. Helminths can be recognized in ultrasound images by their morphology, size, and location. In some cases, the parasite stages are not directly seen by ultrasound, but the lesions caused by them can be easily visualized. Ultrasound imaging is taking on an increasingly important role in the diagnosis, staging, monitoring, and control of parasitic diseases in veterinary medicine. However, it cannot replace the clinical approach and the diagnostic tests commonly used in veterinary parasitology.

## 1. Introduction

Parasites are an important cause of disease in domestic animals. The effective control and treatment of parasitic diseases requires rapid and accurate diagnostic tests which can also serve to monitor the effectiveness of the therapeutic and prophylactic plans. Diagnosis and monitoring of parasitic diseases in live animals can be achieved by several direct and indirect methods, in addition to history, geographic location, and clinical signs. Direct diagnosis is usually established by the detection of the adult parasites or its various stages (eggs, larvae, cysts, and oocysts) in feces, blood, tissue specimens, and other biological fluids (urine, bile, exudate, cerebrospinal fluid, and aqueous humor in the anterior chamber of the eye). Novel direct molecular tests for the identification of parasites have been developed but, at this stage, they are mainly used for research purposes. Indirect diagnosis could be reached by serology-based tools, such as antigen-detection tests and antibody-detection assays [1]. Diagnostic imaging may aid in the diagnosis of parasitic disease as it allows the direct visualization of parasite stages and/or the lesions caused by parasites themselves [2]. Moreover, imaging follow-up examination allows disease progression and response to treatment or vaccination monitoring in live animals. During the last 30 years, ultrasonography (US) has become an essential diagnostic tool in veterinary practice because it is noninvasive and painless [3]. Furthermore, contrary to other advanced diagnostic imaging techniques, such as computed tomography (CT) and magnetic resonance (MR), US does not require sedation of the animals and can be easily performed in the field, thanks to the presence of high-performance battery-powered devices. This allows professionals to screen a large number of animals in a short period of time, providing instantaneous results.

The purpose of this systematic review was to summarize the current knowledge on application of US in diagnosis, staging, and monitoring of helminthic diseases in domestic animals.

## 2. Materials and Methods

A literature search on the PubMed and Scopus databases was carried out between October 2021 and March 2022. Only peer reviewed research papers concerning the application of US in the diagnosis, staging, and monitoring of parasitic diseases in domestic animals, written in English language, and published until 31 March 2022, were included. 

Key words used in the search were:

Ultrasound AND veterinary AND parasitology.
Ultrasonography AND veterinary AND parasitology.
Ultrasound AND (parasites OR parasitology) AND (small animals OR ruminants OR swine OR horses OR equids).
Ultrasound AND veterinary AND (nematodes OR cestodes OR trematodes).
Ultrasound AND animals AND (nematodes OR cestodes OR trematodes).

**Figure 1:**
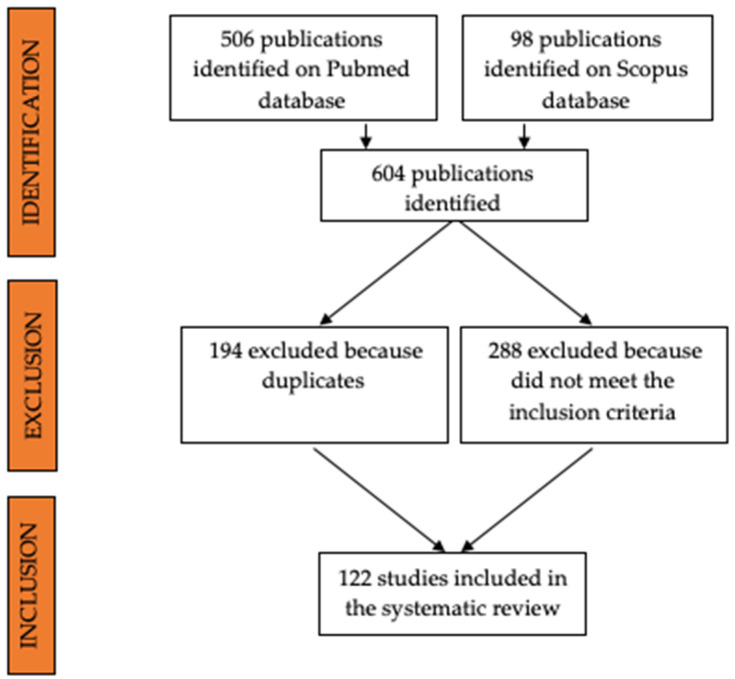
Flow diagram showing the selection process of papers included in this systematic review.

All the papers concerning unicellular parasites, wild animals, non-domestic experimental animals, or ex vivo or in vitro applications of ultrasonography were excluded from the review. Article abstracts were exported into a reference manager software (Mendeley Desktop version 1.19.8) and duplicates were removed (Figure 1). 

## 3. Results

A total of 122 studies met the inclusion criteria. Among the selected papers 47% concerned nematodes, 37% cestodes, and 16% trematodes. The selected publications were about the application of US in animals affected by parasites of the genus *Echinococcus* (21%), *Dirofilaria* (19%), *Dioctophyme* (9.5%), *Angiostrongylus* (8.5%), *Fasciola* and *Mesocestoides* (6% each), *Coenurus* and *Heterobilhaarzia* (5% each), *Toxocara* and *Cysticercus* (4% each), *Parascaris, Aelurostrongylus*, *Onchocerca*, *Spirocerca, Platynosomum* (2% each), *Metorchis*, *Schistosoma*, and *Troglostrongylus* (1% each).

### 3.1. Cestodes

Cystic echinococcosis (CE) caused by *Echinococcus granulosus sensu lato* (*E. granulosus s.l*) is one of the most frequent parasitic diseases in the Mediterranean basin, Eastern Europe, Central Asia, China, North Africa, and South America, and is considered one of the most widespread anthropozoonoses in the world [4,5,6,7,8]. The life cycle of *E. granulosus s.l.* involves domestic and wild carnivores as definitive hosts that harbor the adult stages, and wild and domestic mammals as intermediate hosts, in which the larval stages usually develop in the liver and the lungs. Hydatidosis in livestock animals is an economic issue because it determines the qualitative and quantitative reduction in animal production [9]. 

**Figure 2:**
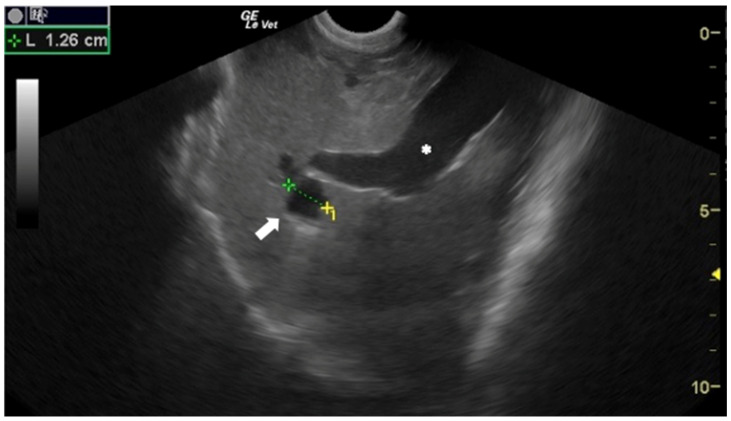
Sheep liver with a unilocular, rounded, anechoic *E. granulosus* cyst (arrow). Portal vein (*). Courtesy of the Veterinary Teaching Hospital, University of Sassari (Italy).

Although in human medicine the earliest use of US for the diagnosis of CE dates to the 1970s [10,11], in veterinary medicine it was introduced much more recently. The US appearance of *E. granulosus s.l.* cysts depends on their stage [12]. Active and fertile cysts appear as hypo/anechoic, rounded, unilocular structures (Figure 2).

In 1996, Maxson and others evaluated the use of US to study the prevalence of hydatidosis in sheep and goats in Kenya. In this study, 260 sheep and 320 goats underwent an US scan of the liver and the right lung, and a subsequent postmortem examination of the positive animals. Ultrasonography identified hydatid cysts in 24 (9.2%) sheep and in 8 (2.5%) goats. Postmortem examination confirmed the diagnosis in 18 (6.9%) sheep and in 5 (1.5%) goats. According to the authors the false positives were mostly due to the presence of *Taenia hydatigena* (*T. hydatigena*) cysts in the liver [13]. 

In 2001, a group of Argentinian researchers evaluated the usefulness of abdominal US in identifying *E. granulosus* cysts in the liver and kidneys of sheep. In this study, a total of 142 sheep coming from endemic (*n* = 102) and non-endemic areas (*n* = 40) underwent US examination. Twenty-two sheep were examined in a slaughterhouse just before being slaughtered and consecutively underwent postmortem examination. Among the other 120 sheep, only the animals that tested positive on liver US were subjected to a necroscopic examination. On US, anechoic cyst-like structures were found in the liver of six animals of 2 years of age or older coming from endemic areas, and the diagnosis of CE was confirmed by the postmortem examination in these six animals. No hepatic cysts were found in the sheep from the non-endemic areas and no cysts were found in the kidneys of the 142 sheep. Authors stated that no shearing was required before US examination and the entire procedure required approximately 4 min per animal [14]. 

A Tunisian study, published in 2007 by Lahmar et al., was the first to apply the ultrasonographic classification of hydatid cysts, used in human medicine, to small ruminants. The authors scanned the liver of 1039 sheep between the ages of 1 and 14. The sheep were clipped on the right side, and the examination was performed with the animals in left lateral recumbency [15]. All the hepatic cysts, detected by US, were classified based on their morphology and size into five types [16] that correspond in the World Health Organization (WHO) classification [12] to the types CE1, CE3, CE2,CE4 and CE5, respectively (Table 1). More recently, the same US classification was also used by Dore et al. [17] and Borriello et al. [18].

The results of this study indicated a total prevalence of hepatic CE of 40.42% with higher values in animals older than 8 years (62.44%) and lower in animals aged between 1 and 2 years (20.66%). The hydatid cysts were found mainly in the right lobe of the liver (near the portal vein), in the hepatic veins, and in the gallbladder. Necropsy, performed on 18 animals, revealed that US had not been able to identify cysts located in the left lobe of the liver [15]. The authors argued that this was due to the poor accessibility of the US to that area. The same group of authors evaluated the efficacy of the US-guided intracystic injection of dipeptide methyl ester on the protoscolices of viable *E. granulosus* cysts in sheep. Twenty-one viable hydatid liver cysts (17 Type I, 3 Type II and 1 Type III) of 15 live sheep were treated by the puncture-aspiration and subsequent injection-reaspiration of dipeptide methyl ester, under US guidance. A viability test of the protoscolices was immediately performed, using the hydatid fluid taken, and follow-up of the treated cysts was done monthly, by US, until euthanasia. Results showed that after the injection of the drug and the reaspiration of the liquid, all protoscolices were killed in less than 15 min, all treated cysts underwent a reduction in size, degeneration of the content, and calcification of the wall [19]. The US-guided sampling of cystic fluid, used for diagnostic purposes, has also been described in cattle and buffalo in 2017 [20].

The diagnostic accuracy of US in diagnosing CE was first evaluated by Maxson et al. in 1998 [21]. They measured the sensitivity and specificity of US in detecting CE in the liver and right lung of sheep and goats. US and necroscopic examinations were performed at an abattoir in Kenya on 16 sheep and 284 goats, presented for slaughter. The comparison between the results of the US and those of the postmortem examination reported a sensitivity of 54.36%, a specificity of 97.64%, a predictive positive value (PPV) of 80.64%, and a negative predictive value (NPV) of 92.19%. Authors stated that some of the false positives were due to the presence of hepatic *T. hydatigena* cysts, and that most of the false negatives were due to the pulmonary and cranial liver location of the hydatid cysts [21]. In Maxson’s studies, the right thorax and the abdomen were clipped before the US scan and approximately 30 animals per hour were examined [13,21]. In 2014, an Italian study, reported a sensitivity of 88.7% and a specificity of 75.9% with a PPV 81.8% and a NPV of 84.6% of US in detecting CE in the liver of sheep [17]. In this study, the operators did not shear the animals and examined about 12 sheep per hour (5 min/animal). A third study, published by Hussein and Elrashidy in 2014, described the diagnostic performance of US in diagnosing CE in sheep. Ultrasound examination images were compared to necropsy findings in ten animals. The results showed a sensitivity of 80%, a specificity of 100%, a PPV of 100%, and NPV of 83% [22]. More recently, a group of researchers of the University of Naples (Italy) assessed the diagnostic performance of US in diagnosing CE in 172 sheep of different breeds reporting a sensitivity of 91%, a specificity of 80%, a PPV of 80%, and NPV of 91% [18]. The diagnostic accuracy of US in diagnosing CE in sheep and goats, reported by the aforementioned studies, is described in detail in Table 2.

Hussein and Erlashidy’s study also described the US findings of the ovine liver during CE [22]. The authors reported that infected animals presented hepatomegaly, hyperechoic and heterogeneous liver parenchyma, decreased portal vein diameter, increased dorsal margin and reduced diameter of the gallbladder [22]. In a study published by Sagkan-Ozturk et al., US was used as an intra vitam diagnostic test to diagnose CE in a group of nine sheep and 17 lambs in which oxidative/antioxidative status and liver function were also studied [23].

The examined scientific literature shows that US is a valid diagnostic tool for CE in ruminants and that it can be used as an intra vitam screening method in control programs for this pathology in endemic areas [24].

Dogs and cats can act as accidental intermediate hosts of the larval stages of *E. granulosus* and *Echinococcus multilocularis* (*E. multilocularis*). Ultrasonographic description of peritoneal CE has been reported in cats [25,26,27,28]. Generally, feline CE presents as multiple cystic cavities with a hyperechoic wall and anechoic content attached to the peritoneum or free in the peritoneal cavity. The US features of lesions caused by the larval stage of *E. multilocularis* have been described in the liver, prostate, and pancreas of dogs [29,30,31,32,33,34,35]. The most common US findings in alveolar echinococcosis in dogs were: hepatomegaly, presence of multiple hepatic large cavitary masses with fluid echogenic content delimited by ill-defined hyperechoic and irregular walls [29].

Coenurosis is a parasitic disease caused by the larval stage of *Taenia multiceps* which affects various ruminant species particularly sheep and goats and occasionally humans. The adult stage of this tapeworm develops in the small intestine of wild and domestic canids, and the larval stage can develop mainly in the central nervous system of ungulates and humans [36,37]. Although CT and MR imaging are the gold standard in diagnosing ovine cerebral coenurosis [38,39], these techniques are not affordable for use in farm animal practice. On the contrary, US, despite the various limitations on its use over bone tissue, is more accessible as it is cheaper, does not require anesthesia of the examined animals, and can be performed in the field [40]. The use of US in locating the cerebral coenurosis cysts in small ruminants has been reported in three studies [41,42,43]. Doherty et al. described the US localization of *Coenurus cerebralis* during surgery by applying the transducer, covered with a sterile surgical glove, directly on the brain’s surface after removal of the bone tissue [42]. In contrast, Athar et al. in 2018 and Biswas in 2013, described the use of transnuchal US, using the rarefied bone portion as an acoustic window, in the localization of cerebral cysts in sheep and goats [41,43].

The US aspects of retrobulbar cystic coenurosis by *Taenia serialis* have been reported in a rabbit and in a chinchilla. In both cases, the cysts were described as retrobulbar hypoechoic masses surrounded by thin hyperechoic walls [44,45]. 

Cysticercosis caused by *Cysticercus tenuicollis* (*C. tenuicollis*) is a metacestode infection that affects several ungulate species. It is caused by the larval stage of *T. hydatigena*, an intestinal tapeworm in dogs and wild canids. In the intermediate host, the mature cysticerci are usually found in the omentum, mesentery, and peritoneum, and less frequently in the pleura and pericardium. 

**Figure 3:**
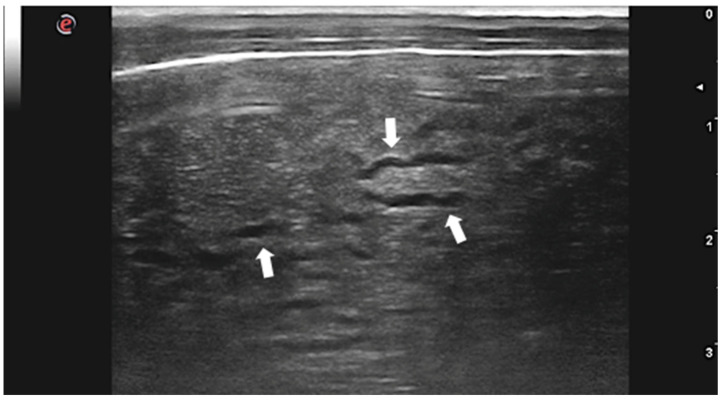
Lamb liver with intraparenchymal anechoic tracts produced by *C. tenuicollis* migration (arrows). Courtesy of the Veterinary Teaching Hospital, University of Sassari (Italy).

Although *C. tenuicollis* infection is quite common in sheep farming, there is only one study describing the US appearance of the hepatic lesions caused by larval migration in lambs with acute cysticercosis [46]. Livers of affected animals presented rounded margins and a thickened, irregular, and hyperechoic surface. Hepatic lesions appeared as heterogeneous areas crossed by numerous, irregular, and anechoic tracts ranging from 1 to 2 cm in length and 0.1 to 0.2 cm in width (Figure 3). Several superficial and intraparenchymal cystic structures were also visualized by US. The presence of lesions and larvae was confirmed by anatomopathological examination, and *C. tenuicollis* was identified by morphological and molecular characterization of isolates.

Ultrasound has been shown to be a valuable diagnostic tool of *Cysticercus cellulosae* cysticercosis in live pigs. Herrera-Garcia et al., in 2007, described the US appearance of the larval form of *Taenia solium* (*T. solium*) in the eye, tongue, masseter, neck, and proximal region of thoracic and pelvic limbs of affected pigs. Cysticerci appeared as fluid filled, oval or rounded cysts containing an elongated echogenic structure that corresponded to the scolex [47].

The diagnostic performance of US in diagnosing cysticercosis in pigs has been evaluated by Flecker et al. in 2017. A total of 152 pigs, seropositive for presence of antibodies against *T. solium* cysts, underwent US examination and subsequent necropsy. The medial aspects of both fore limbs and hind limbs were scanned, by US, for viable cysts which were defined as cystic structures with clearly delineated borders containing clear vesicular fluid and a central opacity. Authors required a minimum of two viable cysts, identified by US, to consider the result positive. Complete necropsy was then performed by systematically dissecting the whole carcass of all pigs and identifying any viable cysts. The results of the study evidenced that US has a specificity of 90% and a sensitivity of 100% in diagnosing porcine cysticercosis in pigs with ≥100 viable cysts identified by necropsy [48]. 

The US appearance of mesenteric cysticercosis by *Taenia pisiformis* in a rabbit has recently been described. The cysts presented as rounded, anechoic structures embedded in the mesenteric fat which was focally mildly hyperechoic. The cysts measured ≤ 9 mm in diameter, were septated, and contained a well-defined, eccentric, hyperechoic nodule adherent to the inner wall [49].

**Figure 4:**
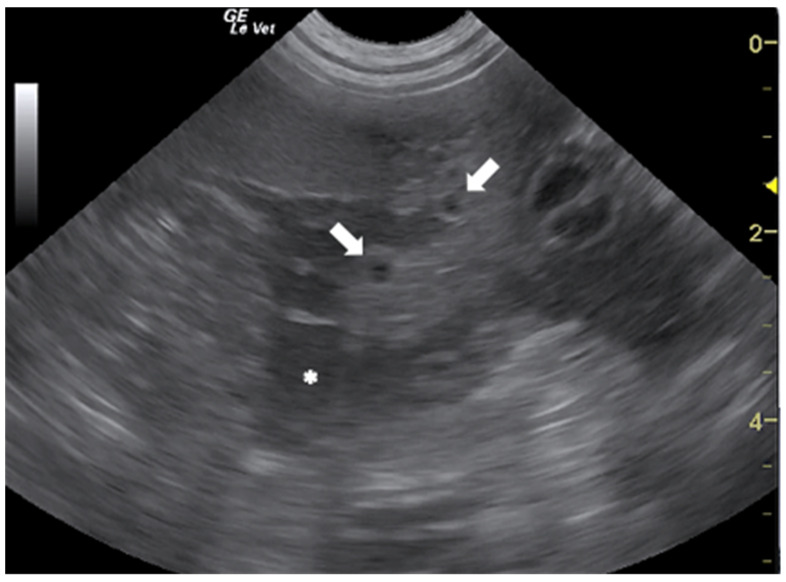
Ascitic effusion (*) and anechoic cystic structures in the omentum (arrows) in a dog with peritoneal mesocestodiasis. Courtesy of the Veterinary Teaching Hospital, University of Sassari (Italy).

In the literature, there are several papers describing the US findings of peritoneal and pleural mesocestodiasis by *Mesocestoides* spp. in dogs and cats [50,51,52,53,54,55]. The most common US features, reported in dogs with peritoneal mesocestodiasis are characterized by the presence of ascitic effusion with multiple anechoic cystic structures (≤2 cm) free in the fluid and disseminated on the surface of abdominal organs and omentum (Figure 4). In all the described cases, the cysts were confirmed to be the acephalic larvae of the parasite [50,51,53,54]. 

In a case report of peritoneal mesocestodiasis in a cat, US evidenced the presence of several non-cystic hypoechoic and elongated structures, characterized by a widened head and thin tail with uniform internal architecture, localized on the serosal surface of abdominal organs. The presence of abdominal effusion was not reported [56]. The different US features, reported in peritoneal mesocestodiasis, are due to the possibility of dogs and cats to host two morphologically distinct forms of the parasite: the cystic acephalic larvae, described in dogs, or the tetrathyridia larvae, described in a cat [51]. Both forms are capable of asexual reproduction and can cause severe peritonitis. 

### 3.2. Nematodes

The first US description of nematodes in small animals dates to 1988, when a group of clinicians from the University of Illinois described the appearance of adult worms of *Dirofilaria immitis* (*D. immitis*) in the right heart of dogs with heartworm disease [57]. Badertscher et al. described the adult parasites as two parallel echogenic lines separated by a hypo-anechoic region. Moreover, they demonstrated that images of worms fixed in formalin had an identical appearance. 

**Figure 5:**
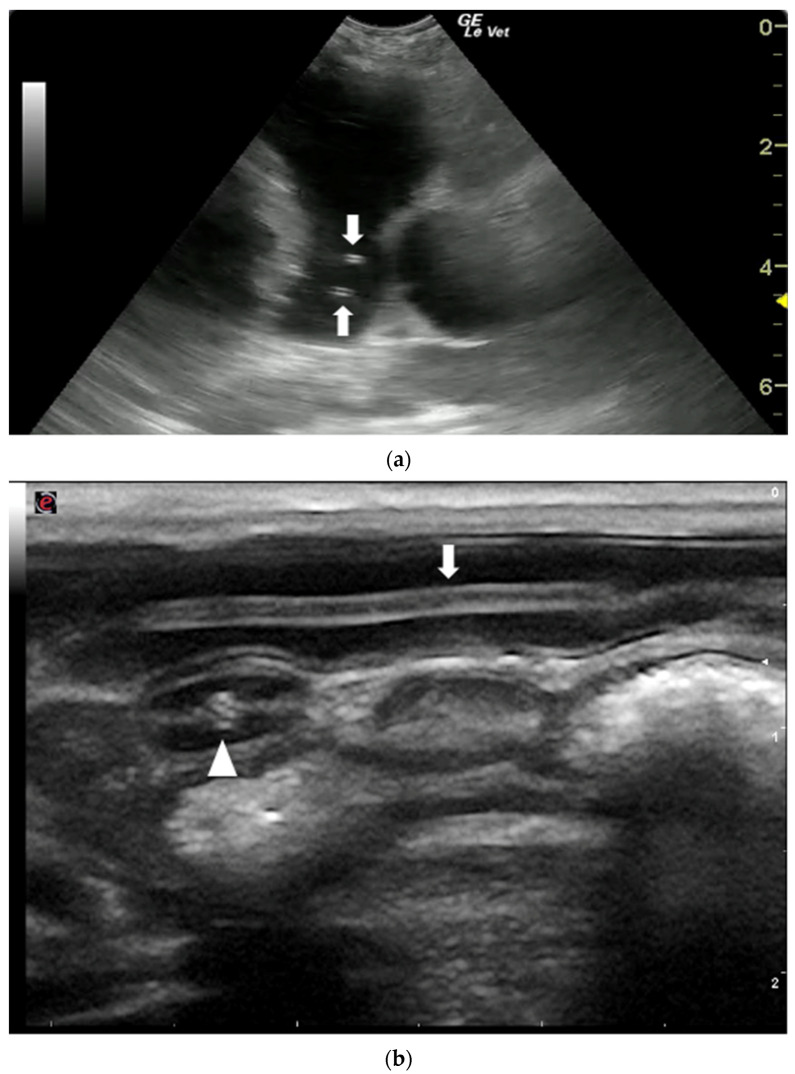
Ultrasonographic appearance of nematodes. (**a**) *T. canis* worms in the small intestine of a puppy, longitudinal (arrow) and transversal section (arrowhead) of the parasite. (**b**) Sections of adult *D. immitis* worms in the pulmonary artery of a dog (arrows). Courtesy of the Veterinary Teaching Hospital, University of Sassari (Italy).

The US appearance of nematodes is due to the structure of the parasite which, in longitudinal section, appears as a linear “train track“ structure composed of two parallel hyperechoic lines, that represent the cuticle, separated by a narrow hypo/anechoic line constituted by the alimentary canal. In cross section, they have a “donut” appearance characterized by a hyperechoic circular wall with a hypo-anechoic center [58,59,60,61,62,63] (Figure 5).

In the second half of the 1990s, the use of US in veterinary medicine in western countries experienced an exponential increase which, associated with the continuous improvement in image resolution, led to the multiplication of echocardiographic diagnosis of heartworm disease, also in cats [64,65,66,67,68,69,70] and ferrets [71]. In these two species, the scarce and transient microfilariaemia, and the low parasitic burden of infected animals reduce the sensitivity of the hematologic analysis, making US a fundamental diagnostic test for this disease [67,72,73,74]. 

Echocardiography was used for assessing the heartworm burden in experimentally infected cats [66]. The results of Atkins’ study evidenced a sensitivity of 95% and a specificity of 63% of US in detecting heartworm infection in cats. According to the authors, the worm burden estimated with cardiac US correlated well with necropsy counts, especially in heavy infections (>11 adult worms) which are uncommon in natural infections. 

Adult nematodes of *D. immitis* have rarely been visualized by US in the systemic arterial circulation of dogs [75,76,77] and cats [78]. Systemic arterial dirofilariasis is a rare manifestation of *D. immitis* infection that results from unusual migration of the parasites into the peripheral arterial circulation. The proposed mechanisms for systemic arteriolar migration include aberrant migration of the L5 worms or relocation of adult worms via a right to left cardiac shunt. Clinical signs of systemic arterial heartworm disease depend on the parasite’s localization and on the degree of ischemia caused by the presence of worms and associated thrombosis. They include pelvic limb lameness and paresis, hemorrhagic diarrhea, interdigital necrosis, and exercise intolerance [75,77,78]. Ultrasound imaging is essential in detecting aberrant localizations of adult heartworms because negative Knott’s microfilarial and heartworm antigen tests do not rule out systemic dirofilariasis [75]. Ultrasonographic diagnosis of heartworm disease involves direct visualization of the adult parasite in the vascular and/or cardiac lumen [57,79]. This is made possible by the size of the adult parasites which can reach 10–30 cm in length. 

Ultrasonography can be useful not only for direct visualization of adult *D. immitis* parasites but also for US-guided parasite removal procedures and for detecting signs of pulmonary arterial hypertension secondary to heartworm disease [56,80,81,82,83,84]. 

Pulmonary hypertension can also be secondary to other respiratory parasitosis such as angiostrongyliasis by *Angiostrongylus vasorum* (*A. vasorum*) in dogs [85,86,87,88,89,90], aelurostrongylosis by *Aelurostrongylus abstrusus* or troglostrongylosis by *Troglostrongylus brevior* in cats [91,92,93], and lungworms in small ruminants [94]. However, these adult nematodes cannot be directly visualized by US due to their small size (10–15 mm) and localization in the respiratory system [95,96]. Nevertheless, an ectopic migration of *A. vasorum* larvae into the vitreal body of the right eye has been recently visualized by ocular US in a dog [97].

Recently, a study conducted in Italy has shown that the US evidence of subpleural, hypoechoic nodules, with a rounded shape, and a diameter between 2.5–25 mm, mainly located in the caudo-dorsal areas of both lungs, has a sensitivity of 100% and a specificity of 92% in the diagnosis of *A. vasorum* infection in dogs with respiratory symptoms, aged between 4 months and 2 years [98]. The pulmonary lesions, reported in this study, are in accordance with those described previously in dogs with angiostrongyliasis [97,99,100]. The distinctive US appearance of nematodes made it possible to identify the parasitic origin of a subcutaneous nodule in a cat infested with *Dirofilaria repens*, thus excluding that it was a typical feline fibrosarcoma and radically changing the prognosis [101]. 

Ultrasound has been useful in diagnosing nodular lesions caused by nematodes of genus *Onchocerca* and *Spirocerca*. Lia et al. in 2017 described the US aspects of two nodules localized in the right and left metacarpal regions of a horse. Ultrasonographic images of both structures were consistent with a peritendinous localization of a verminous nodule. Subsequent histopathological, morphological, and molecular examinations confirmed that they were *Onchocerca boehmi* parasitic granulomas [102]. Ultrasonography has also been applied in the diagnosis and monitoring of ocular canine onchocercosis by *Onchocerca lupi* (*O. lupi*) [103,104]. The nematodes appeared as hyperechogenic structures located in different ocular regions of the affected dogs: the sclera-corneal junction, the ocular medial rectus muscle, and the retrobulbar space. In one case, the diagnosis was confirmed by morphological and molecular identification of *O. lupi* [103]. The US appearance of a nodular gastric lesion caused by *Spirocerca lupi* (*S. lupi*) in a 2-year-old dog was described in 2014. In this dog, US identified a 1.3 × 2 cm, well defined, hypoechoic, and homogeneous gastric mass which was surgically removed. Diagnosis of spirocercosis was achieved by histopathological, morphological, and molecular examinations [105]. Dogs with *S. lupi* infection have a higher proportion of US evidence of abdominal aortic and celiac arterial wall abnormalities, such as wall thickening, wall irregularities or mineralization, intraluminal thrombus, hyperechogenicity around the celiac artery, and celiac artery aneurism [106].

Ascarids can be easily visualized using US examination. They are roundworms, with a circular section, that live in the small intestine and their dimensions are variable depending on the species. The US pattern of gastrointestinal and biliary ascariasis has been widely described in human medicine since the 1980s [107,108,109,110]. The first US description of intestinal ascaridiosis in small animals was published in 2007 [63]. The parasites were described as structures with two to three linear parallel hyperechogenic lines separated by a hypoechoic center and characterized by undulatory movements. The diagnosis was confirmed by fecal examinations revealing the presence of *Toxocara cati* and *Toxocara canis’* eggs, respectively in a cat and in a dog. 

In 2019, an Italian study assessed the diagnostic performance of abdominal US in diagnosing *T. canis*’ infection in puppies during the prepatent period. The study results demonstrated that US has a sensitivity of 85.4% and a specificity of 100% in diagnosing *T. canis* infections in puppies of 15 days of life, when fecal examinations were still negative [58].

In small animals, roundworms can migrate, although rarely, from the duodenum to the extrahepatic biliary duct and cause biliary obstruction and infection. In these cases US allowed prompt diagnosis and guided the treatment decision [111].

Infection with *Parascaris* spp. is very frequent in foals with less than 12 months of age and massive infections can cause acute small intestine obstruction and death. Ultrasonography is a useful diagnostic imaging modality for documenting small intestinal dilatation and presence of intraluminal ascarid worms [112,113]. In 2016, a group of American researchers validated an US scoring method for transabdominal monitoring of *Parascaris* spp. burdens in foals’ small intestine [61]. The authors scanned three abdominal regions from the foal’s left side: 1. immediately caudal to the xiphoid, 2. midway between the xiphoid and the umbilicus, and 3. immediately cranial to the umbilicus. For each examination, they scored the images’ quality and the ascarid burden visualized in the intestinal lumen. The study compared the US scoring method to the necropsy-confirmed ascarid burden. The results provided evidence that 81% of examinations generated useful images and that the US scoring technique can reliably detect ascarid burdens larger than ten worms [61]. Ultrasound examination can also help clinicians in the diagnosis of intestinal obstruction due to *Toxocara vitulorum* in the calf [114].

The nematode *Diocthophyme renale* (*D. renale*), commonly called “the giant kidney worm”, is the largest known parasitic nematode, infecting domestic animals. The females can reach up to 103 cm long and 0.5–1.2 cm wide, while the males can reach up to 45 cm long and 0.3–0.5 cm wide. *D. renale* reaches maturity in several mammalian species, including humans [115]. Among domestic animals, the nematode is more frequently observed in dogs than in cats, horses, or cattle [116]. In the definitive host, the parasites are located more frequently in the kidneys, the right kidney more often than the left one, causing a gradual compressive atrophy of the renal parenchyma [116]. However, adult worms have been reported in the abdominal and thoracic cavity, retroperitoneal space, uterus, ovary, mammary gland, urinary bladder, urethra, subcutaneous tissues, abdominal muscles, and lymph nodes [116,117,118,119,120]. In dogs and cats, the diagnosis by renal US is commonly performed [117,118,120,121,122,123,124,125]. Ultrasonographic features included nephromegaly of the parasitized kidney whose pelvis, or the entire organ, appears occupied by multiple ring-like (transversal section) or tubular-shaped (longitudinal section) structures of 5 to 10 mm in diameter. Both sections of the parasite were characterized by two echogenic walls separated by an anechoic central area. When the parasites occupied the whole kidney, there was a complete loss of the internal renal architecture [118,120,122,123,124,125]. 

### 3.3. Trematodes

Infestations caused by *Fasciola hepatica* (*F. hepatica*) affect the hepatobiliary system and occur in two stages: the hepatic stage (acute and invasive) and the biliary stage (chronic and obstructive). In the acute phase, the immature parasites pass through the mucosa of the small intestine up to the peritoneal cavity and migrate into the liver causing massive destruction of the parenchyma and severe bleeding. The chronic stage, which develops once adult flukes have established within the biliary ducts, is characterized by chronic cholangitis and cholestasis [126]. Ultrasonographic findings during experimentally induced fascioliasis by *F. hepatica* infection in rabbits and in sheep have been described in 1999 and in 2003, respectively [127,128]. In both studies, the US findings during the parenchymal phase (7–8 weeks post infection) of the disease were characterized by the presence of hypoechoic lesions that progressed to hyperechoic areas irregularly distributed within the hepatic parenchyma. In the biliary stage (9–10 weeks post infection), the US images were more specific, as the biliary duct appeared dilated, tortuous, increased in size, and with moving echogenic parasites within the dilated ducts [39,127].

Scott et al. in 2005 described the US appearance of the liver and the cranial abdomen of 15 lambs (7 months old), with naturally acquired *F. hepatica* infection, during the subacute stage of the disease. The US examination was performed on standing animals, and it was limited to a maximum of 5 min per sheep including skin preparation. Ultrasonographic examination revealed the presence of peritoneal fluid, heterogeneous liver echotexture with multiple irregular 3–10 mm hyperechoic dots disseminated in the parenchyma, and adhesions between small intestine and liver. Post-mortem examination confirmed the US findings, highlighting the severe liver damage caused by immature flukes’ migration, and the widespread adhesions between liver, abdominal wall, diaphragm, and small intestine [129].

The first description of US findings in cattle and buffaloes with chronic hepatic fascioliasis was published in 2012. Sixteen animals (seven cattle and nine buffaloes), symptomatic and positive for *Fasciola* spp. infection, underwent abdominal US examination. A total of ten animals presented gallbladder distension and in seven of them the presence of echogenic sediment was evident. Other US findings included edematous gallbladder wall, dilation of the intra- and extrahepatic bile ducts, bile duct mineralization, heterogeneous and hyperechogenic hepatic parenchyma, and abdominal effusion. US observations were subsequently confirmed by post-mortem examination of two cows and one buffalo [130].

An Egyptian study conducted in 2016–2017 aimed to investigate the association between the US images of the liver of infected cattle and buffaloes and the severity of *Fasciola* spp. infection. Seventy-four animals, that tested positive for *Fasciola* spp. on fecal examination, were subjected to US scan: 38 of them (14 cattle and 24 buffaloes) had a low level of egg count on fecal examination and showed a normal hepatic parenchyma and bile system, while 20 of them (6 cattle and 14 buffaloes) had a moderate level egg count and showed hyperechogenic hepatic parenchyma with multiple echogenic foci and normal bile system. Finally, 16 animals (5 cattle and 11 buffaloes), had a high level of egg load and the US images revealed abdominal and thoracic effusion, hyperechogenic hepatic parenchyma, mineralized bile ducts, and distended gallbladder. The results of the study revealed a good correlation between the hepatobiliary US changes, the fecal egg load, and the clinical manifestations in cattle and buffaloes with fasciolosis [131].

More recently, Alizadeh et al. examined, by US, the liver of 256 sheep with a diagnosis of chronic *F. hepatica* infection. Hepatic US examination was performed on animals, restrained in dorsal and left lateral recumbency, in a period between 5 and 15 min with a low frequency convex probe. According to the results, 55.6% of the sheep had ascites along with multiple subcapsular, small, confluent, and hypoechoic nodules with poorly defined borders. Moreover, in 5.7 and 11.3% of the animals, the bile ducts and gallbladder were affected, respectively, while in 4.8% of them both the liver and the bile ducts were involved [132]. In cases where bile ducts were involved, 11 sheep (61.1%) had duct dilatation without the presence of *F. hepatica* and seven sheep (38.9%) had duct dilatation with parasites. The most common site of injury was in the posterior part of the right liver lobe [132]. A heterogeneous US appearance of the liver parenchyma of an alpaca with acute fascioliasis has recently been reported [133]. 

In infections with *Dicrocoelium dentriticum*, US examination of the liver revealed enlarged and occasionally calcified bile ducts in sheep [17].

Ultrasonography is being increasingly applied to detect and quantify hepatointestinal, hepatosplenic, and genitourinary lesions caused by the trematodes of the genus *Schistosoma* [134]. To better understand the US evolution of hepatic lesions caused by *Schistosoma* infection, in 2003, a group of nine pigs was experimentally infected with 1000 *Schistosoma japonicum* cercariae at 12 weeks of age. The infected pigs underwent liver US examination, slaughtering, gross pathological observation, and collection of parasitological data. Ultrasound, carried out 12 weeks post infection, revealed hepatomegaly, thickening of the portal vessels’ walls, diffuse increase in hepatic echogenicity, and dilatation of the portal vein. At the post-mortem examination, all the alterations were confirmed and a good correlation between the degree of hepatic fibrosis and the hyperechogenicity of the parenchyma was found [135].

The trematode *Heterobilharzia americana* (*H. americana*), the causative agent of canine schistosomiasis, causes granulomatous gastrointestinal and hepatic disease in dogs. Infection occurs through contact with water contaminated by cercariae that, after penetrating the host’s skin, migrate to the lungs and liver. There takes place their sexual maturation [136]. Ultrasonographic findings were peritonitis and multisystemic mineralization characterized by multiple hyperechoic shadowing foci in the liver, pancreas, mesenteric lymph nodes, and intestinal walls [136,137,138,139]. Bilateral nephrolithiasis and urinary bladder sediment were also reported [138,140]. The results of a recent study evidenced that the combination of heterogeneous small intestinal wall layering, particularly the submucosa, and pinpoint hyperechoic foci in the small intestine, liver, or mesenteric lymph nodes was the most reliable sonographic indication of *H. americana* infection in dogs with a PPV of 94% and specificity of 96.4% [139].

*Platynosomum* species are cat-specific parasitic liver trematodes that occupy the hepatic ducts and gallbladder of cats from tropical and subtropical regions of the world [141]. The most common US alterations seen in feline platynosomiasis are not specific and include: sediment in the gallbladder, hyper- or hypoechogenicity of liver parenchyma, hepatomegaly, thickened gallbladder wall, and dilated bile ducts [142,143].

The liver fluke *Metorchis conjunctus* (*M. conjunctus*) is a trematode which infects various fish-eating animals, including dogs, with a high prevalence in Canada [144]. In the definitive host, the adult trematodes migrate up the biliary tree causing hepatic lesions. In the literature, there is only one case report describing the US appearance of liver lesions associated with *M. conjunctus* infection in a dog. These alterations were characterized by multiple liver abscesses, thickened walls, and distension of the intra- and extrahepatic bile ducts, and presence of echogenic sediment in the gallbladder, cystic duct, and common bile duct. Percutaneous US-guided drainage, lavage and, alcoholization of the abscesses was useful to make a diagnosis and to successfully treat the dog [145].

## 4. Discussion

In the present review, we have summarized the current knowledge on the application of US in helminthic diseases of domestic animals. As evidenced by our results, the application of US in diagnosing, staging, and monitoring infections caused by nematodes and cestodes is the most reported in scientific literature with 47% and 37% of the included papers, respectively. 

Nematodes are easily recognized in US images. They are characterized by a linear structure composed of two parallel hyperechoic lines, separated by a narrow inner hypoechoic zone, in longitudinal sections, and a hyperechoic circular wall, with a hypo-anechoic center, in transverse sections (Figure 5). The nematode most commonly described by US in human medicine is *Ascaris lumbricoides* which can be visualized in the intestinal lumen and in the biliary tract of infected patients [60]. In veterinary medicine the species *D. immitis* and *D. renale* are the most easily identifiable due to their size and their localization in the cardiovascular lumen and in the kidneys, respectively. In canine and feline heartworm disease, US is a fundamental test to assess the severity of the disease and, consequently, to guide clinicians in therapeutic decisions. Moreover, US plays an important role in the diagnosis of heartworm disease in cats [67] and of aberrant *D. immitis* localizations in dogs and cats [75,76,77,78]. In both cases, negative Knott’s microfilarial and heartworm antigen tests may occur and, the direct US visualization of the adult parasites allows the disease to be diagnosed. However, in both dogs and cats, the lack of US evidence of parasites in the pulmonary arteries and/or in the right heart does not rule out infection [65,67,82]. Therefore, US images should always be interpreted considering the clinical findings and the results of other diagnostic tests.

Among the cestodes, the cystic larval stages are easily identifiable by US because of their dimension and fluid component. In the case of CE, US also allows assessment of the viability of the larval stages and, according to several authors, the US screening of sheep from endemic areas of hydatidosis, can be considered a reliable technique in epidemiologic surveillance systems and in the evaluation of the results of disease control programs, such as vaccination [14,146,147,148,149]. In human medicine, US remains the first-choice modality in the diagnosis and monitoring of both alveolar and cystic echinococcosis [150,151,152]. The test reported a sensitivity of 100% and a specificity of 95.6% in the diagnosis of human CE [153].

The variability in diagnostic performance of US, reported by different authors in small ruminants CE (Table 2), is probably due to numerous reasons. Firstly, the variety of examined species and breeds could have led to differences in body conformation, size, and weight which could explain the inconsistent ability of US in obtaining images of the whole liver. Secondly, the higher percentage of exclusively pulmonary localization of cysts could have reduced the sensitivity of US in Maxson Sage’s study [21]. In fact, pulmonary cysts are not detected by US unless they are in direct contact with the thoracic wall. Thirdly, the different local prevalence of concomitant parasitic diseases could have caused false positives in the examined animals. Another cause for the discrepancy in the results could be the different US instrumentation used in the reported studies. Maxson et al. used a linear transducer, Dore et al. and Borriello et al. used microconvex probes whereas Hussain et al. used both convex and linear probes [17,18,21,22]. Additionally, the technological progress that US has undergone from 1996 to 2012–2019 could be a further element that explains the increased sensitivity reported by Dore, Hussain and Borriello et al. Finally, the different duration of the US examination: 2 min/animal vs. 5 and 7 min/animal reported by Maxson, Dore and Borriello et al., respectively, might have had an impact on the results [17,18,21,22]. Ultrasonographic examination is also considered a valid intra vitam screening test for porcine cysticercosis in those geographical areas where this pathology represents a health issue for humans and animals [48]. Both CE and *T. solium* cysticercosis are zoonotic parasitic diseases, included in the WHO list of the priority neglected zoonotic diseases for which effective control efforts are needed [154]. Based on these considerations, US could be regarded as a diagnostic tool that contributes to integrated control systems that aim to eradicate these diseases [24].

As evidenced by our results, the trematodes were the least described by US (16%) probably due to their small size and morphology which make them not easily identifiable in US images. However, the technological improvement and the growing diffusion of US in the veterinary field, will certainly lead to a greater research interest in fluke infections in domestic animals.

When the parasites cannot be directly identified by US, the method can detect and quantify the specific lesions caused by the parasites themselves in the host organism. In cases of acute *F. hepatica* or *C. tenuicollis* infection in lambs, US could represent a fundamental intra vitam diagnostic test for initiating therapy and reducing mortality [46,129]. However, it is important to point out that focal, or diffuse parenchymal lesions can also be caused by various non-parasitic diseases such as vascular, infective, traumatic, autoimmune, metabolic, iatrogenic, neoplastic, congenital, and degenerative illness. This is also true for cystic structures that can also be caused by hematomas, abscesses, neoplasia, and other lesions of a non-parasitic origin. For this reason, US imaging should always be evaluated very carefully and integrated with clinical and laboratory results before making a diagnosis of parasitic disease.

Ultrasound is increasingly applied in veterinary medicine [2] due to many reasons. Firstly, the versatility of the method which finds application in reproduction, orthopedics, and internal medicine. Secondly, the availability of using small portable US systems powered by batteries and sold at an affordable price. Thirdly, the rapidity by which diagnostic results are obtained, and finally, the possibility of examining non-sedated animals in the field. The main limitation of US is the high dependence on operator skills and experience. Undoubtedly, soon there will be a remarkable improvement in the definition of US images that will allow veterinary clinicians to recognize parasites and parasitic lesions that currently can only be viewed by direct visualization during necropsy. Furthermore, we will see an increasing use of US not only in the diagnosis, but also in the epidemiological surveillance and in the monitoring of therapeutic and prophylactic protocols applied as treatment and prevention of parasitic diseases of animals. However, although US can undoubtedly help with the diagnosis, monitoring, and control of parasitic diseases, it cannot replace the clinical approach and diagnostic methods used in veterinary parasitology.

## 5. Conclusions

The scientific literature examined shows that US is a valid diagnostic tool for helminthic disease in domestic animals. The versatility of the method and the constant technological improvement of the equipment are allowing a wide diffusion of this diagnostic imaging technique in the field of veterinary parasitology. In our opinion, ultrasound should be considered an auxiliary method that can be integrated with epidemiological data, clinical findings, and laboratory diagnosis of parasitic diseases in domestic animals.

## Figures and Tables

**Table 1:** Ultrasonographic World Health Organization classification of *Echinococcus* cysts.

Gharby Scheme Type	Gharby US Description	WHO Classification
Type I: pure fluid collection	Unilocular round shape, well-defined walls, anechoic content	CE1 Active and fertile
Type II: fluid collection with a split wall	Unilocular, less rounded shape, well defined contour, anechoic content, and presence of “split wall” sign.	CE3 Transitional stage
Type III: fluid collection with septa	Multilocular, rounded or oval shape, well defined contour, fluid collection divided by septa.	CE2 Active and fertile
Type IV: heterogeneous echo patterns	Roughly rounded masses with irregular contour and variable echo pattern: 1. hypoechoic with irregular echoes, 2. solid, hyperechoic, and not shadowing, 3. intermediate including hypoechoic and hyperechoic structures	CE4 Inactive
Type V: reflecting thick walls	Structures with hyperechoic and shadowing contour	CE5 Inactive

**Table 2:** The diagnostic accuracy of ultrasonography in diagnosing cystic echinococcosis in sheep and goats reported by different studies.

Author	Maxson Sage et al. [21]	Dore et al. [17]	Hussein et al. [22]	Borriello et al. [18]
Year of study	1996	2012	2011–2013	2017–2019
Location	Kiserian, Kenya	Sardinia, Italy	Asyut, Egypt	Southern Italy
Animals	16 sheep of I.B. 284 goats of I.B.	129 Sarda sheep	22 Baladi sheep	172 sheep of different breeds
Wool shearing	Yes	No	Yes	Yes
Probes	L 3.5 MHz	MC 8–11 MHz	L and C 3.5, 5, 8 MHz	MC 6–10 MHz
Duration of US	2 min/animal	5 min/animal	NR	7.1 min/animal
Examined organs	Liver and right lung	Liver	Liver	Liver
Postmortem exam	All animals	All animals	10 animals	All animals
Only pulmonary cysts (%)	15.2	3.9	0	1.7
Sensitivity (%)	54	89	80	91
Specificity (%)	98	76	100	80
PPV (%)	81	82	100	80
NPV (%)	92	85	83	91

IB, indigenous breeds; L, linear; MC, microconvex; C, convex; US, ultrasound; NR, not reported; PPV, predictive positive value; NPV, negative predictive value.

## Data Availability

Not applicable.
